# Increases in Retrograde Injury Signaling Complex-Related Transcripts in Central Axons following Injury

**DOI:** 10.1155/2016/3572506

**Published:** 2016-10-25

**Authors:** Gunja K. Pathak, Hannah Ornstein, Helim Aranda-Espinoza, Amy J. Karlsson, Sameer B. Shah

**Affiliations:** ^1^Fischell Department of Bioengineering, University of Maryland, College Park, MD, USA; ^2^Department of Chemical and Biomolecular Engineering, University of Maryland, College Park, MD, USA; ^3^Departments of Orthopaedic Surgery and Bioengineering, University of California, San Diego, La Jolla, CA, USA

## Abstract

Axons in the peripheral nervous system respond to injury by activating retrograde injury signaling (RIS) pathways, which promote local axonal protein synthesis (LPS) and neuronal regeneration. RIS is also initiated following injury of neurons in the central nervous system (CNS). However, regulation of the localization of axonal mRNA required for LPS is not well understood. We used a hippocampal explant system to probe the regulation of axonal levels of RIS-associated transcripts following axonal injury. Axonal levels of importin *β*1 and RanBP1 were elevated biphasically at 1 and 24 hrs after axotomy. Transcript levels for *β*-actin, a prototypic axonally synthesized protein, were similarly elevated. Our data suggest differential regulation of axonal transcripts. At 1 hr after injury, deployment of actinomycin revealed that RanBP1, but not importin *β*1, requires de novo mRNA synthesis. At 24 hrs after injury, use of importazole revealed that the second wave of increased axonal mRNA levels required importin *β*-mediated nuclear import. We also observed increased importin *β*1 axonal protein levels at 1 and 6 hrs after injury. RanBP1 levels and vimentin levels fluctuated but were unchanged at 3 and 6 hrs after injury. This study revealed temporally complex regulation of axonal transcript levels, and it has implications for understanding neuronal response to injury in the CNS.

## 1. Introduction

The poor regenerative capability of the central nervous system (CNS), compared to the peripheral nervous system (PNS), limits recovery from a number of traumatic and degenerative conditions. On the other hand, central neuroregeneration has been observed in limited contexts (e.g., [1, 2]), indicating a need to better understand mechanisms underlying regenerative capacity.

A key advance in understanding mechanisms underlying the robustness of PNS regeneration was the identification and characterization of a retrograde injury signaling (RIS) pathway, which is hypothesized to require transport of injury signals from the injury site to the cell body. Details of this pathway in peripheral neurons have been well summarized in several reviews [3–7]. Briefly, rapid ion influx at the injury site generates a rapid response that propagates retrogradely to provide the first indication of lesion events [3, 8–10]. A slower component of RIS results in dynein-dependent transport of an injury-induced signaling complex from the site of injury to the nucleus [3, 7, 11]. Importantly, injury also induces local axonal translation of several proteins required for RIS complex transport, including importin *β*1 (karyopherin *β*1), RanBP1, and vimentin [3–5, 7, 12, 13].

Importin *β*1, among its diverse roles [14–17], is a key node in RIS pathways, as its knockout attenuates transcriptional responses to nerve injury and delays functional recovery in vivo [6]. Interestingly, recent evidence suggests that importin *β*1 may also play a role in central neuronal regeneration. Importin *β*1-associated STAT3 signaling molecules were transported retrogradely after injury of hippocampal neurons, but only when the importin-STAT association was intact [18, 19].

Despite these compelling advances, several key gaps remain in understanding RIS mechanisms in central neurons. A key unknown is whether and by what mechanisms levels of importin *β*1 and other RIS-associated transcripts are altered locally in the axon in response to injury. The goal of this study was to perform an initial examination of importin *β*1-dependent RIS mechanisms in the CNS, through the use of a new hippocampal explant system [20], which enables examination of axonal mRNA and protein expression independent of neuronal cell bodies. Our results suggest a biphasic axonal response, in which levels of several axonal transcripts, including those associated with RIS, increase rapidly in axons after injury. Importin *β*1-dependent activity at the nucleus then appears to modulate a second wave of RIS-associated transcripts, which are likely to further support axonal outgrowth.

## 2. Methods

### 2.1. Explant Culture

All animal protocols were approved by the University of Maryland Institutional Animal Care and Use Committee (IACUC).

Details and validation of the explant culture system, including assessment of axonal purity, have been published [20]. Briefly, C57/Black 6 mouse neonates (P1) were euthanized. Brains were harvested and maintained in cold Hank's Balanced Salt Solution (HBSS). Hippocampi were detached from the surrounding tissue and seeded on lysine-coated glass cover slips in Neurobasal media supplemented with 2% B-27. Media were changed carefully every 3 days, so as not to dislodge the explant. All cells were maintained at 37°C and 5% CO_2_. Detached explants or fragmented hippocampi were discarded.

### 2.2. Axon Isolation and Inclusion Criteria

**Figure 1:**
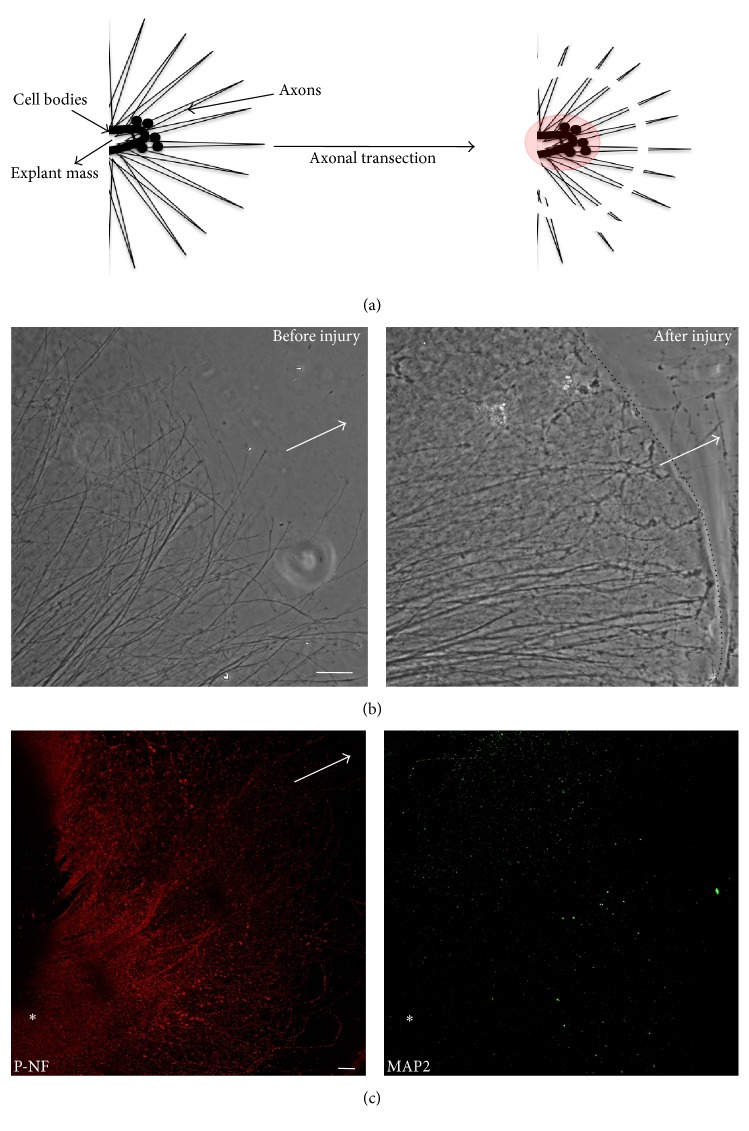
Hippocampal explant injury model. (a) Schematic of axonal injury, performed 2/3 distance away from the edge of the explant. Axons were collected proximal and distal to the injury site. Cell bodies (shaded red) were excluded from collection. (b) Bright-field image illustrating long, narrow axons before and after axonal injury. (c) Double-label immunofluorescence of SMI-31 (red) and MAP2 (green) showed robust axonal outgrowth and very few or no dendrites in the same region (green nonspecific binding). Arrow indicates direction of outgrowth. Asterisk (*∗*) indicates explant mass. Additional details of explant system are provided in [20]. Bars are 75 *μ*m.

Explants were allowed to grow for seven days, after which axons were severed with a needle at a distance ~2/3 of the longest axons away from the explant edge. Injured or noninjured axons were collected using appropriate lysis buffer and a micropipette, through careful observation under a light microscope, and aspiration perpendicular to the axons. To avoid any cell body and dendritic contamination, we avoided regions at the explant edge (Figure 1(a)). For severed axons, tissue was collected both proximal and distal to the injury site, to enable comparison with corresponding control axons. We performed additional analysis on each sample to assess the exclusive axonal nature of the preparation, as described previously [20]. For RT-PCR assays, we confirmed the absence of *γ*-actin mRNA by PCR, which resides only in the soma but is restricted from axons [21–23]. For protein assays, we confirmed the absence of NeuN, a neuron-specific nuclear, and thus axon-excluded, protein by immunoblotting [24]. Any samples that were contaminated with cell body markers were not used for further analysis; ~30% of samples used for PCR and ~10% of the samples used for Western blot experiments were rejected due to cell body contamination.

### 2.3. Axonal Transcript Levels

Control or injured axonal samples were collected at 1, 6, 15, and 24 hours after injury. For detection of axonal transcripts, ~100 ng of RNA from axons was used as a template for reverse transcription (RT) with M-MLV Reverse Transcriptase (Invitrogen, Carlsbad, CA) and an oligo (dT) primer at 90°C for 1 h. The RT reactions were diluted 10-fold and used for transcript-specific PCR. For primer sequence, we used Primer 3 tool based on specific nucleotide sequence found on PubMed. Primer sequences used for PCR are outlined in Table 1. Negative controls were performed on each sample, and they consisted of RNA processed without the addition of reverse transcriptase. For quantitative RT-PCR, the control and sample RT reactions above were amplified using the Thermocycler detection system (Bio-Rad, Hercules, CA). These reactions were performed using the SsoFast EvaGreen Supermix (Bio-Rad, Hercules, CA) for all transcripts. All control and samples were assayed in triplicate for four independent experiments. Thermal cycling was initiated with an initial denaturation at 50°C for 2 min and 95°C for 10 min followed by 40 cycles at 95°C for 15 s and 60°C for 1 min. Relative levels of individual transcripts were calculated by normalizing to the CSF1 control using a comparative threshold (Ct) value method. Briefly, the Ct for each transcript was determined using the automatic Ct algorithm of the My IQ software to calculate the optimal baseline range and threshold values. Individual ΔCt values were then determined by subtracting the CSF1 Ct value from the individual transcript Ct values. From this, the calculation of ΔΔCt was determined by subtracting ΔCt sample (injury to the axons) from ΔCt control (no injury to axon). The fold difference was then expressed as 2^−ΔΔCt^, with ΔΔCt + SD and ΔΔCt − SD, where SD is the standard deviation attained from Ct values as described by Livak and Schmittgen in [25]. Transcript levels are expressed relative to ΔΔCt values of the control (uninjured) axons. As some transcripts were expressed at very low levels in axons, primers were validated in whole explant lysate.

### 2.4. Actinomycin *D* Treatment

Actinomycin D (AMD) dose was set at 5 *μ*g/mL, a dose that rapidly and persistently suppresses all classes of transcription in mammalian cells, based on ^3^H-uridine incorporation and reduction of a variety of transcripts differing in GC-richness of sequence and length [26]. Treatment duration was varied from 1 to 3 hrs to test AMD efficacy in explants. As AMD treatment resulted in substantial reduction of multiple transcripts within two hours, explants were treated with this dose for two hours before being injured (or not, for controls). Samples were collected one hour after injury and RT-PCR was performed as above, with AMD treated uninjured axons used as controls.

### 2.5. Inhibiting Nuclear Transport

Importazole (IPZ; Sigma-Aldrich, St. Louis, MO) has been well-characterized in nonneuronal cells [27]. We followed a similar approach to characterizing inhibition of importin-mediated nuclear import in our system. Explants were transfected 12 hrs prior to IPZ treatment with NFAT-GFP expression plasmid (pKW520, a kind gift from Dr. Karsten Weis), using Effectene Transfection Reagent (Qiagen, Valencia, CA) as per manufacturer's instructions. All transfections took place in Neurobasal media supplemented with 2% B-27 (Life Technologies).

IPZ was used at concentrations of 18 *μ*M for up to 24 hours, supplying fresh media with IPZ every 12 hours. For controls, fresh media without IPZ were used. At 22 hours (1.5 hours prior to 24-hour treatment), ionomycin was added at 15 *μ*M to induce intracellular calcium influx and trigger nuclear import. To assess import, cells were fixed with 4% formaldehyde prior to fluorescence microscopy. DNA was visualized with 1 *μ*g/mL Hoechst dye. For quantification, 100–200 cells for each condition were analyzed and nuclear accumulation of NFAT-GFP was assessed using Image J.

### 2.6. Cell Viability

To test cell viability, after transfection and IPZ and ionomycin treatments, cultures were washed three times and assessed using live/dead reagent (Invitrogen, Grand Island, NY), as per manufacturer's protocol. Explants were incubated for 20 minutes, washed, and imaged via an inverted Nikon TE-2000E microscope. Cell viability of explants containing 0.1 to 0.4% of DMSO was performed as a control.

### 2.7. Immunofluorescence and Imaging

Hippocampal explants were fixed with 4% paraformaldehyde in PBS for 10 minutes and rinsed with PBS three times. Following permeabilization with 0.2% Triton X-100 in PBS, the cells were blocked with 10% fetal goat serum and 3% BSA for 30 minutes. 1 : 1000 dilution of SMI-31 (Abcam Inc., Cambridge, MA) and/or 1 : 500 dilution of MAP2 (Abcam Inc., Cambridge, MA) in BSA was applied for 1 hour at room temperature, followed by three washes in PBS. Fluorescently labeled secondary antibody (AlexaFluor-488 and AlexaFluor-594, Life Technologies) was then applied for 1 hr at 37°C, followed again by three washes in PBS.

### 2.8. Immunoblotting

After 1, 3, and 6 hours of injury, control (uninjured) axons and positive control (whole explant) samples were lysed using NP40 lysis buffer mixed with protease inhibitor (Fisher Scientific, Houston, TX) and phosphatase inhibitor (Roche Diagnostics, Indianapolis, IN, USA). The homogenate was further lysed in liquid nitrogen, and supernatant was stored at −80°C. Protein concentration was measured using a bicinchoninic acid (BCA) protein assay kit (Pierce; Thermo Fisher Scientific, Rockford, IL, USA). An equal amount of protein (60 *μ*g) was loaded into each lane, run on 4–15% Mini-Protean Precast gel (Bio-Rad, Hercules, CA), and transferred to PVDF membranes (Millipore, Billerica, MA). The membranes were blocked overnight using casein-blocking buffer (Vector Lab, Burlingame, CA) and incubated for 2 h at RT either with RanBP1 antibody (diluted 1 : 100; Santa Cruz, Dallas, TX) or with vimentin antibody (diluted 1 : 100; Abcam, Cambridge, MA). Alternately, membranes were incubated for 35 minutes at RT with importin *β*1 antibody (diluted 1 : 300; Abcam, Cambridge, MA), GAPDH (diluted 1 : 60000; Fitzgerald, Acton, MA), or NeuN (diluted 1 : 500; Millipore, Billerica, MA). Appropriate secondary antibodies and DuoLux Chemiluminescent were used for detection following the protocol provided in Vectastain ABC-AmP kit (Vector Lab, Burlingame, CA). The membranes were detected using ChemiDoc™ XRS+ (Bio-Rad, Hercules, CA) and analyzed using Image Lab software (Bio-Rad, Hercules, CA), which accounted for regional background subtraction. Quantification was performed at subsaturation levels for each blot. All data were normalized to GAPDH levels.

### 2.9. Microscopy

Bright-field and phase contrast imaging were performed on an inverted TE-2000E microscope (Nikon, Melville, NY) outfitted with Lumen-PRO2000 (Prior Scientific, Rockland, MA) illumination system. A custom built chamber (Precision Plastics, Beltsville, MD) maintained temperature, humidity, and CO_2_ levels during imaging. Leica SP5X confocal microscope (Buffalo Grove, IL) was used for explant imaging. The confocal system was equipped with multiple laser lines, including a 405 diode, an Argon laser (458, 476, 488, 496, and 514 nm), and a white light laser (470–670 nm in 1 nm increments); samples were imaged using either a 10x or 40x objective with filtering appropriate to visualize Hoechst (Excitation 350 nm and Emission 451 nm), SMI-31 (Excitation 579 nm and Emission 599 nm), and MAP2 (490 nm and Emission 530 nm) markers.

### 2.10. Statistics and Sample Sizes

All PCR experiments were assayed in triplicate for at least three independent experiments (axonal injury experiments: *N* = 3 for 1 and 24 hrs and *N* = 4 for 6 and 15 hrs time points; AMD and IPZ experiments: *N* = 3, except for Ranb1, in which only a single sample amplified this transcript, possibly due to IPZ effects on both control and injured populations, unrelated to injury response). Quantitative PCR data were compared statistically using the relative expression software tool (REST©, Qiagen, Valencia, CA), as described previously [28]. Type I error *α* was set to 0.05. For importazole characterization, 100–200 cells were quantified from two different explants for each condition, control, and IPZ treatment for 12 hrs and 24 hrs. Two-way Student's* t*-test, with Bonferroni's adjustment for multiple comparisons, was used to compare means. For immunoblots, 3–5 independent experiments were performed per group at each time point. Due to lack of normality within data sets, data were reciprocally transformed and means of transformed data were compared using 1-way ANOVA followed by Dunnett's test. For all experiments, type I error *α* was set to 0.05.

## 3. Results

### 3.1. Explant Model for Examining Isolated Hippocampal Axons

To examine the axonal expression and regulation of transcripts involved in CNS RIS, we developed a mouse hippocampal explant system, which enables injury and analysis of isolated axons [20]. For this study, we cultured P1 explants for seven days and cut the axons on the seventh day to study axonal response to injury at time points up to 24 hours (Figures 1(a) and 1(b)). Uninjured axons at the same time point were used as controls. Immunofluorescence evaluation of an axonal marker, phosphorylated neurofilament (SMI-31), and a dendritic marker, microtubule-associated protein 2 (MAP-2A), confirmed the axonal nature of long projections from the explant (Figure 1(c); cf. [20]). The exclusion of nuclear or cell body markers was assessed in the axonal lysate of each sample, and contaminated samples were excluded from analysis. Transcripts of interest present and absent in cell lysate and axons are shown in Table 2.

### 3.2. Influence of Axotomy on Axonal mRNA Expression

**Figure 2:**
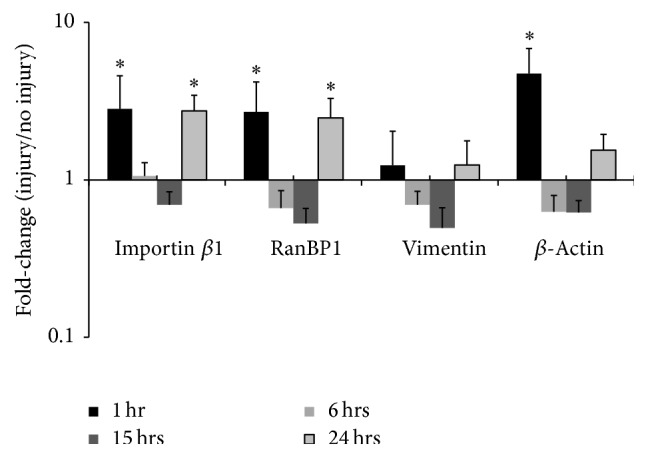
Axotomy results in biphasic axonal increase of RIS-associated transcript levels. Real-time RT-PCR was used to quantify levels of putative RIS-associated mRNAs and *β*-actin in axons after axotomy. All values are displayed relative to uninjured controls. Significant increases in importin *β*1, RanBP1, and *β*-actin mRNA levels at 1 hour after axotomy, suppression at 6 and 15 hrs after axotomy, and increases again at 24 hours after axotomy for importin *β*1 and RanBP1 were observed. No significant changes were observed in vimentin transcript levels. Error bars represent standard deviations. *∗* indicates significant difference from control (*p* < 0.05) based on pairwise fixed allocation test. *N* = 3 for 1 and 24 hrs, and *N* = 4 for 6 and 15 hrs.

Based on the retrograde transport of RIS complexes observed in both the PNS and CNS [3, 4, 7, 12, 18, 19], we hypothesized that levels of transcripts encoding RIS complex components would too be increased in axons. To test this hypothesis, we first performed RT-PCR at 1, 6, 15, and 24 hrs after injury. Injury caused a significant increase in axonal levels of importin *β*1 (2.82 ± 1.7, 4.5; *p* < 0.002) and RanBP1 (2.70 ± 1.5, 4.9; *p* < 0.009), but not vimentin (*p* = 0.46), 1 hr after axotomy relative to control (no injury). Transcripts encoding *β*-actin, which is a well-described locally synthesized protein not believed to play a role in RIS, also significantly increased 1 hour after axotomy compared to controls (4.73 ± 2.1, 10.6; *p* < 0.003), serving as a “positive control” (Figure 2). Axonal levels of all four transcripts were not significantly different between injured and uninjured axons 6–15 hours after axotomy (*p* > 0.05). Interestingly, 24 hrs after axotomy, we observed a second wave of increased levels of importin *β*1 (2.74 ± 0.7, 10.6; *p* < 0.06) and RanBP1 (2.48 ± 0.8, 7.5; *p* < 0.05) mRNA compared to controls. *β*-Actin mRNA levels also trended higher at 24 hours (*p* = 0.45), but differences with controls did not reach statistical significance (Figure 2). These results suggest a biphasic elevation in putative RIS-associated transcripts.

### 3.3. Influence of Transcriptional Inhibition on Axonal mRNA Expression

**Figure 3:**
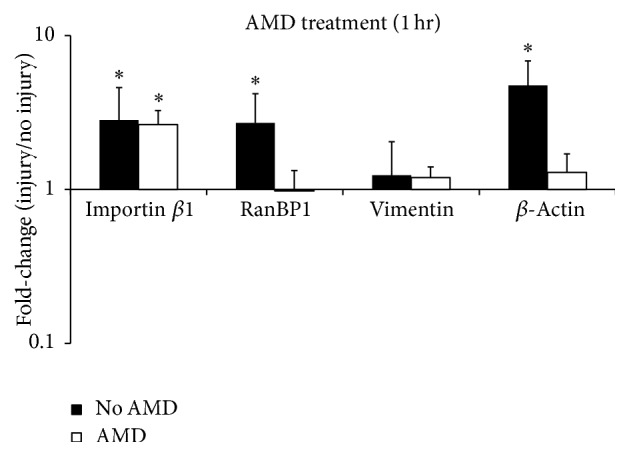
Inhibition of transcription results in differential axonal transcriptional changes. Real-time RT-PCR was used to measure levels of axonal mRNA in injured hippocampal axons after inhibiting transcription with AMD. All values are displayed relative to uninjured controls. Results indicate persistent elevation of importin *β*1 mRNA levels after transcriptional inhibition but not RanBP1, vimentin, and *β*-actin. Black bars (previously graphed in Figure 2, 1 hr) indicate untreated axons and are included for clarity of comparison. Error bars represent standard deviations. *∗* indicates significant difference of AMD treated and untreated cells from their corresponding controls (*p* < 0.05) based on pairwise fixed allocation test. *N* = 3 for all groups.

The above results support the hypothesis that an injury-induced signal causes elevation in axonal levels of importin *β*1, RanBP1, and *β*-actin at an early stage, within 1 hr after axotomy. We next assessed whether this increase in transcript levels required de novo transcription in the cell body. We used the well-characterized antibiotic actinomycin D (AMD) to inhibit transcription and performed RT-PCR to measure changes in transcript levels at 1 hr after injury. To identify conditions under which AMD reduced transcript levels in our culture system, we quantified total mRNA in response to a range of times. A dose of 5 mg/mL of AMD for two hours resulted in ~50% total transcript reduction, and so this dosage was used for subsequent experiments. AMD treatment suppressed the injury-induced increase in mRNA levels of RanBP1 (0.98 ± 0.34, 2.74) and *β*-actin (1.29 ± 0.42, 3.79) observed in untreated cells, suggesting that these genes were newly transcribed and transcripts were rapidly recruited to axons. However, surprisingly, AMD treatment did not inhibit the injury-induced increase in axonal importin *β*1 mRNA levels (2.64 ± 0.60, 11.5; *p* < 0.001). These results suggest that increased axonal importin *β*1 transcript levels reflect contributions from preexisting transcript populations in the cell body or axons proximal to the level of harvest (Figure 3; black bars are replotted from Figure 2, for comparison).

### 3.4. Influence of Axotomy on Axonal Protein Levels

**Figure 4:**
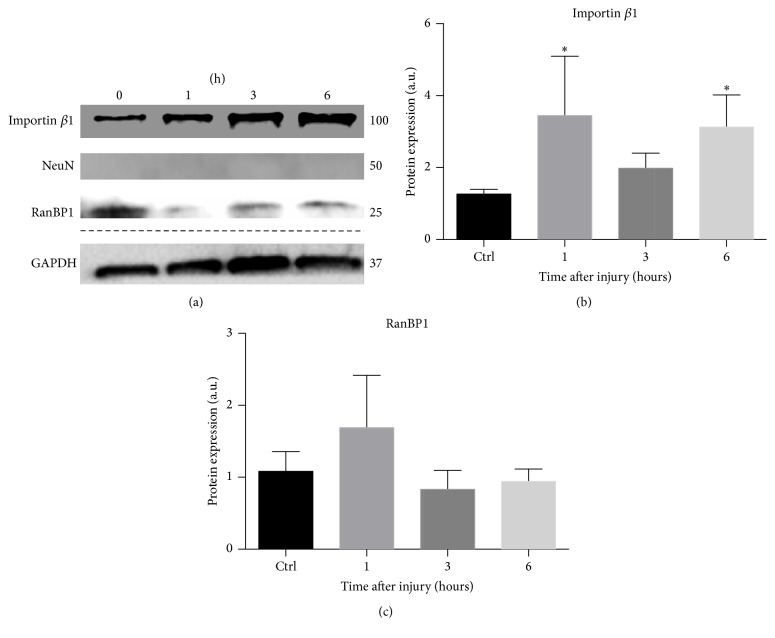
Importin *β*1 protein levels are significantly increased at 1 and 6 hours of injury. (a) Representative Western blots of several RIS-associated proteins (normalized to GAPDH). Note that GAPDH levels vary slightly across time points. Western blots also indicate exclusion of NeuN protein from axonal population. (b) Quantification of relative importin *β*1 protein expression. (c) Quantification of relative RanBP1 protein expression. Data are represented as mean ± SEM. Effect of time on expression was assessed using one-way repeated measures ANOVA, and mean values at each time point were compared to control post hoc using Dunnett's test. *∗* indicates significant difference from control (*p* < 0.05); untransformed data are plotted; however, values were reciprocally transformed prior to ANOVA testing, to meet the requirement of normality. Transformed values are shown in Table 3.

To test whether and over what time frame early increases in transcript levels ultimately resulted in changes in protein expression and thus the possible formation of RIS, we explored changes in axonal levels of RIS proteins in response to injury. We evaluated the expression of importin *β*1, RanBP1, and vimentin at 0 hrs, 1 hr, 3 hrs, and 6 hrs after axotomy. As was also the case for mRNA levels, each RIS protein responded uniquely to injury. ANOVA revealed a significant effect of time on importin *β*1 levels (*p* < 0.01); consistent with local axonal translation (though not excluding some contribution from the cell body), post hoc testing revealed that importin *β*1 levels increased significantly at 1 hour (62% increase, *p* < 0.05) and again at 6 hours (84% increase, *p* < 0.005) but not at 3 hours (Figure 4). RanBP1 protein levels fluctuated with time but maintained levels comparable to control at all time points (Figure 4). Vimentin levels also did not appear to change with time; however, results were too variable to be formally compared with sufficient power (data not shown). Reciprocally transformed data used to make appropriate statistical comparisons are provided in Table 3.

### 3.5. Influence of Importin *β*-Mediated Nuclear Import on Axonal mRNA Expression

**Figure 5:**
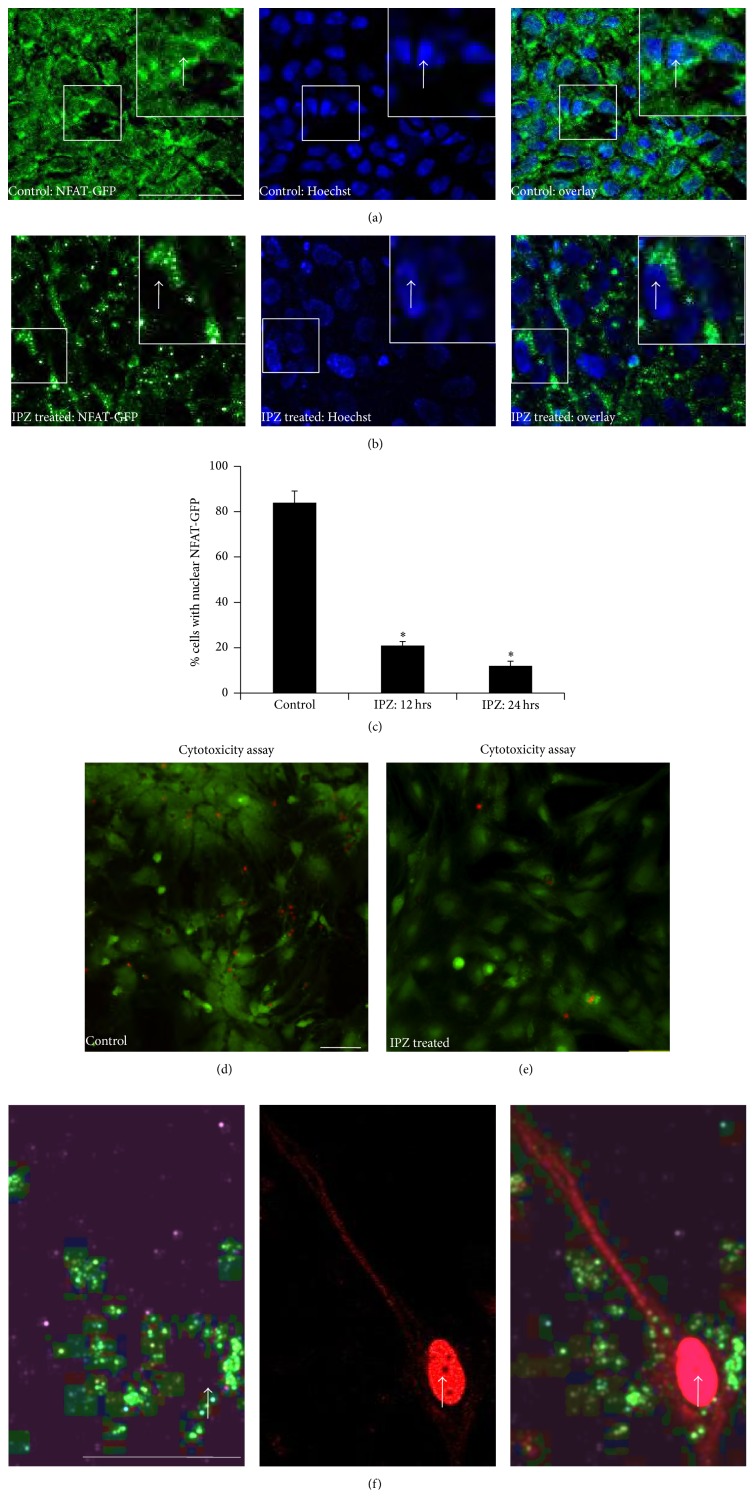
Importazole blocks importin *β*-mediated nuclear import in hippocampal cells. (a) Control cells expressing NFAT-GFP were treated with ionomycin to induce nuclear import of NFAT-GFP. NFAT-GFP localizes within nucleus (Hoechst, arrows) and surrounding regions (overlay, arrows). Inset is expanded for more clear visualization. (b) Cells expressing NFAT-GFP were treated with 18 *μ*M importazole prior to treatment with ionomycin, and nuclear localization was compared to controls in the presence of ionomycin (quantified in c). NFAT-GFP is excluded from nucleus (Hoechst, arrows) but retains extranuclear localization (overlay, arrows). Inset is expanded for more clear visualization. (c) Percentage of cells with nuclear NFAT-GFP. 150 or more cells were counted under each condition. Student's* t*-test with Bonferroni's correction ^*∗*^ *p* < 0.01. (d–e) Live/dead assay of either (d) control or (e) IPZ treated cells treated with ionomycin after transfection shows no toxicity (red) and high viability (green) in both control and treated cells. (f) Given the possibility that only nonneuronal cells were affected by IPZ, we tested whether cells expressing NFAT-GFP were immunolabeled with the axonal marker (SMI-31, red) after IPZ treatment. SMI-31 positive cells indeed expressed NFAT-GFP, thus indicating that neuronal cells were capable of being affected by IPZ treatment. NFAT-GFP signal is observed in the proximal axons. Arrows indicate absence of GFP signal within the nucleus. SMI-31 also cross-reacts with nuclear intermediate filaments, thus resulting in red-labeled nuclei. GFP images have been contrast-enhanced for visualization purposes. Bar is 50 *μ*m. Values represent mean ± SEM.

Having established an increase in both importin *β* transcript and protein at 1 and 6 hours, we next tested whether importin *β*1-mediated nuclear import was required to transcribe new importin *β*, RanBP1, and *β*-actin mRNA observed in the second phase of increased transcript levels at 24 hrs. To minimize nonspecific perturbation of the diverse functional roles of importin *β*1, we specifically disrupted nuclear import mediated via importin *β* using importazole (IPZ). IPZ's role in disrupting importin *β*-mediated nuclear import in nonneuronal cells was extensively characterized [27]. However, IPZ inhibition of nuclear import in an explant system and specifically in neurons has not yet been characterized. We therefore tested for such a role by tracking the localization of the transcription factor NFAT fused to GFP (NFAT-GFP) in response to IPZ treatment. Studies in nonneuronal cells have revealed that NFAT-GFP shuttles between the nucleus and cytoplasm in calcium regulated manner and is imported by importin *α*/*β* and exported by CRM1 [29–32]. As expected, at steady state, NFAT-GFP was predominately cytoplasmic in cells within and at the edge of the explant. Also, as expected, an increase in cytoplasmic calcium induced by ionomycin led to accumulation of NFAT-GFP in the nucleus (Figure 5(a)).

Explants were then treated with 18 *μ*m IPZ for 24 hrs followed by 1.5 hrs of ionomycin treatment in the continued presence of IPZ. In stark contrast to control cells, there was no import of NFAT-GFP in IPZ treated cells (Figures 5(b) and 5(c)); specifically, IPZ treatment resulted in nuclear exclusion of NFAT-GFP in 75% of cells after 12 hrs (*p* < 0.015) and 86% of cells after 24 hrs (*p* < 0.003) (Figure 5(c)), demonstrating effectiveness of IPZ. Because explants contain both neuronal and nonneuronal cells, we performed immunofluorescence labeling with SMI-31 (an axonal marker) to verify that NFAT-GFP was indeed expressed in neuronal cells, and thus neuronal cells were among the cells potentially affected by IPZ (Figure 5(f)). Importantly, transfection and IPZ and ionomycin treatment caused minimal cytotoxicity. Following IPZ and ionomycin treatment of cells expressing NFAT-GFP, we performed a live/dead assay, which indicated over 86% survival of cells treated with DMSO (control), as well as transfected cells treated with IPZ followed by ionomycin treatment (Figures 5(d) and 5(e)).

**Figure 6:**
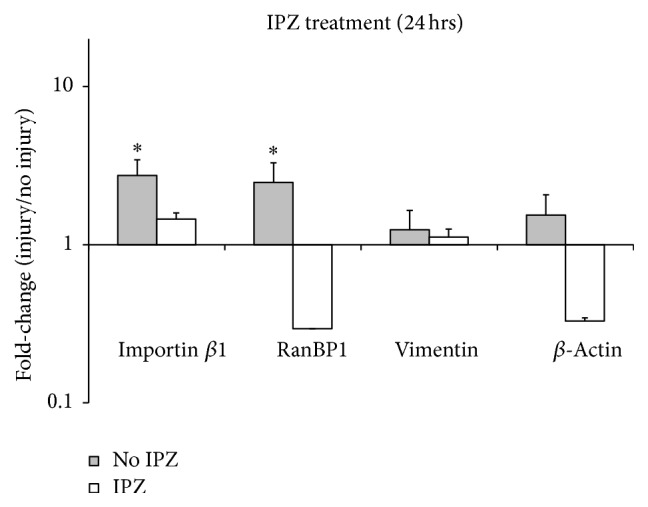
Inhibition of nuclear transport results in downregulation of axonal transcriptional changes. Real-time RT-PCR was used to measure levels of axonal mRNA in injured hippocampal axons after inhibiting nuclear transport with IPZ. IPZ treatment suppresses elevated importin *β*1, RanBP1, vimentin, and *β*-actin mRNA levels (white bars). Grey bars (previously graphed in Figure 2, 24 hrs) indicate untreated axons and are included for clarity of comparison. Error bars represent the SD. *∗* indicates significant difference of IPZ treated and untreated cells from their corresponding controls (*p* < 0.05) based on pairwise fixed allocation test. *N* = 3 for all groups, except for Ranb1 (see Section 2).

We then evaluated changes in axonal mRNA levels 24 hrs after injury followed by IPZ treatment. IPZ treatment suppressed the second wave of increased axonal mRNA for all RIS-related and *β*-actin transcripts, as indicated by the lack of significant changes in axonal mRNA levels of all transcripts after IPZ treatment at 24 hrs after injury relative to control (Figure 6; grey bars are replotted from Figure 2, for comparison). When viewed in combination with the transcriptional and translational upregulation of several key RIS proteins at earlier time points, these results support a model whereby RIS feedback mechanism is required for secondary amplification of both RIS-associated and unassociated mRNA at later time points.

## 4. Discussion

In this study, we examined axonal levels of RIS-associated transcripts, which will ultimately be translated to activate RIS pathways. Our findings suggest that axotomy results in elevation of axonal mRNA, at 1 and 24 hours after injury. Regulation of this biphasic response appears transcript-specific at the early time point, with importin *β*-mediated nuclear import required for the second wave of increased axonal transcript levels for all transcripts.

### 4.1. Biphasic Elevation in mRNA Levels after Axotomy

Inhibition of protein synthesis in axons significantly impairs growth cone activity and axonal extension [33, 34], emphasizing the importance of local protein synthesis for regeneration. Among the many axonally synthesized proteins, translation of importin *β*1, RanBP1, and/or vimentin following axonal injury suggests a role for local protein synthesis in RIS. In particular, local translation of RanBP1 following injury results in RanGTP dissociation from importins, allowing binding of newly synthesized importin *β* to importin *α* and dynein-bound RIS complexes that are transported to neuronal cell bodies [3, 4, 13, 19].

To execute these retrograde signaling pathways, required transcripts must be in place beforehand or must be rapidly recruited to the site of injury [35]. The observed rapid increase in mRNA transcripts within the axon implies rapid localization to the injury site (Figure 2). This response is consistent with the rapid retrograde propagation of an electrophysiological response, possibly mediated by calcium, that provides the first indication of a lesion event [3, 7, 8, 10, 11, 36]. On the other hand, electrical activity alone is not sufficient to initiate regeneration [12, 37], and thus our observed secondary elevation in mRNA transcripts 24 hours after injury is consistent with both RIS and the requirement for an additional signal for effective regeneration. The dip in axonal transcript levels at time points between 1 and 24 hours, as opposed to steadily increasing or stably increased levels, also supports the notion of two different mechanisms for transcript recruitment to the axon.

### 4.2. Specificity and Differential Regulation of Axonal Transcript Levels: Early Injury Response

A number of transcripts have been shown to alter their axonal levels following axotomy of central neurons [35]. As expected, mRNA levels for *β*-actin, whose local synthesis has been extensively characterized, increased after axotomy, serving as a sort of positive control. Consistent with RIS, we also observed rapid increases in both importin *β* and RanBP1 transcript levels but not vimentin. Transcriptional inhibition also revealed differences in the response of evaluated transcripts. The early increase in axonal levels of RanBP1 and *β*-actin transcripts, like GAP-43, depended on newly synthesized mRNAs (Figure 3; [38, 39]). Conversely, importin *β*, like several other proteins, such as CGRP, moved into axons [38, 40], indicating an additional nontranscriptional contribution to axonal synthetic capacity.

Differential regulation of importin *β*1 transcript levels compared to RanBP1 (or vimentin) may in part be a consequence of the functional diversity of importin *β*. In addition to nuclear transport in the cell body, such roles for importin *β* include quality control of ER proteins [17], assembly of mitotic spindles and centrosomes [14, 15], and learning-related plasticity [41]. On the other hand, importin isoform localization is dictated by its 3′ UTR, with short 3′ UTR variant of importin more prominent in cell bodies and longer variant more prominent in axons [6]. Thus, any preexisting recruitable pool of importin *β*1 mRNA must be predesignated for axonal localization. Candidates for such a transport-ready pool are ribonucleoprotein particles (RNPs), which can migrate to axons and dendrites in response to a given stimulus [42, 43]. Such activity has been noted in response to a variety of stimuli, for example, zipcode binding protein 1- (ZBP1-) mediated axonal transport of *β*-actin mRNA following the application of neurotrophins to chick cortical neurons [44, 45]. The identity of zipcode-like mRNA binding proteins that regulate axonal transport of importin *β*1 is yet unknown. However, recent CLIP-Seq data suggests that fused in sarcoma (FUS) and serine arginine-rich splicing factor 1 and factor 2 (SRSF1 and SRSF2) are strong candidates for such a role, based on high numbers of target sites (starBase 2.0; [46, 47]).

### 4.3. A Role for Importin *β* in Regulating Axonal Levels of mRNAs: Delayed Injury Response

Several lines of evidence suggest that importin *β* plays an important, even essential, role in the response to axonal injury. In peripheral neurons, importin-associated RIS complexes transport signaling proteins, including transcription factors such as JNK, Erk, ATF2, and ATF3, from the injury site [4, 5, 48], and the depletion of importin *β* results in suppressed gene transcription and delayed functional recovery following nerve injury [6]. Importin *β* also stimulates axotomy-induced axonogenesis in the CNS, in part by transporting the transcription factor STAT3 [18, 19].

Our data support an important role for importin *β*1 after injury and reveal novel and intriguing temporal dynamics of importin-mediated neuronal response. Based on RIS complex transport rates, transcription rates, and mRNA transport rates [49, 50], it is less likely that importin *β*-mediated nuclear import plays a role in transcriptional changes at 1 hr after axotomy. Though we cannot exclude a rapid signal (e.g., an electrical signal) triggering an initial wave of nuclear import mediated by existing pools of importin *β* in the cell body, insufficient time would have elapsed for the transport of newly synthesized importin *β* into the cell body, import processes to occur, and transcripts to appreciably accumulate. However, our data indicate that importin *β* plays a critical role in the secondary wave of axonal transcript elevation upon injury. Increased importin *β*1 expression over 6 hours (Figure 4) suggests that early increases in importin *β*1 transcript are translated. In addition, axonal elevation of importin *β*1 and RanBP1 transcripts was suppressed following specific pharmacological inhibition of nuclear import, with other importin functions, including RIS complex formation, presumably intact (Figure 6). Together, these data raise the possibility that rapidly synthesized importin *β*1 could feed back to further upregulate the axonal localization of RIS-associated transcripts.

It is important to note that we did not suppress nuclear import (IPZ treatment) at early time points. As a consequence, we do not have insight into the time frame over which nuclear import contributed to upregulation of RIS-associated transcription, nor can we unequivocally state that nuclear import was not involved in the early phase of transcriptional activation. It is possible that suppression of nuclear import and transcription (i.e., AMD and IPZ treatment) at earlier time points may be superposed to additionally inactivate RIS-associated transcriptional pathways. These are limitations of our study. Conversely, we also did not combinatorially suppress transcriptional and nuclear import pathways at the 24-hour time point; as nuclear import precedes transcriptional upregulation, we would expect such an experiment to be uninterpretable. Thus, though overall transcription of RIS-associated proteins dips at intermediate time points, we cannot conclude whether and when pathways contributing to initial transcriptional increases are turned off. Finally, it will be interesting to evaluate the persistence of these putative mechanisms at later time points in future studies.

### 4.4. RIS in the CNS versus PNS

Our work points to interesting similarities and differences between RIS in the PNS and CNS. On one hand, we also observe that axonal levels of the RIS-associated transcripts importin *β*1 and RanBP1 indeed increase following central axonal injury, and importin *β* plays a key role in regulating this increase. This is consistent with the observation in optic neurons (retinal ganglion cells) that hyperactivated STAT3, which interacts with importin *β*, enhanced the axonal regenerative response [51, 52]. Our observed increase in axonal levels of importin *β*1 mRNA after 6 hrs of axotomy (Figure 4) is also similar to that in the PNS, with maximum increases observed 6–8 hrs after axotomy [4]. A recent study by Ohara and colleagues suggested that, unlike our results, several axonal proteins, including importin *β*1, slightly increased 10 mins after axotomy of dissociated cortical neurons, before returning to control levels 1 hr after injury [19]. However, in their study, isolated axons were harvested only from regions distal to the injury site, which were presumably degenerating, and axonal regions proximal to the injury site were not separated from cell bodies [18, 19], precluding direct comparison with our results. Additionally, no further quantification was performed beyond 1 hr.

On the other hand, in contrast to studies in the PNS, which show a gradual increase in axonal protein levels of vimentin and RanBP1 over period of 6 hrs after axotomy in the PNS [5, 13], we observed neither. In fact, our study on CNS neurons indicated unchanged vimentin transcript levels over 24 hours (Figure 2) and no significant change in vimentin or RanBP1 proteins within 6 hrs after axotomy (Figure 4). While, in the PNS, calpain-cleaved vimentin binds to phosphorylated Erks (pErk), linking pErk to dynein via importin *β*1 [5], this regulatory pathway does not appear to be conserved in our CNS model. Our observed RanBP1 axonal levels also differed from observations in the PNS studies. It is not clear why axonal levels of RanBP1 were unchanged after injury; the fact that protein levels did not rise and fall with transcript levels suggests a temporal decoupling of these processes for this protein.

## 5. Conclusions

**Figure 7:**
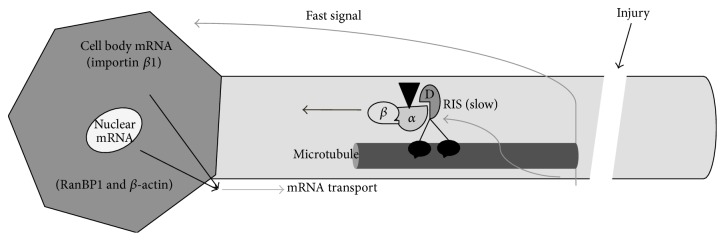
Schematic representation of biphasic axonal response to injury. Upon injury, a fast signal and slower RIS complex transported signal differentially regulate the levels of several transcripts. Changes in axonal protein levels occur over period of 6 hrs, suggesting the dynamic formation of RIS complexes. We posit that the transported RIS complex, via importin *β*-mediated nuclear import, triggers the delayed/second wave of transcriptional changes in the axon.

Our observed biphasic increase in axonal transcript levels suggests tightly and differentially regulated control of local protein synthesis in hippocampal neurons after injury, including a key role for importin *β*-mediated nuclear import (Figure 7). While our focus was on the regulation of RIS-associated transcripts, additional questions regarding RIS signaling, local protein synthesis, and, ultimately, axonal outgrowth in CNS axons remain, including the exact set of transcription factors responsible for de novo mRNA synthesis and how acute transcriptional regulation impacts long-term neuronal outgrowth. Our findings confirm intrinsic regenerative capabilities in the CNS and have important implications for exploiting RIS and local protein synthetic pathways towards enhanced CNS repair.

## Figures and Tables

**Table 1:** Primer pairs for PCR.

Transcript	Primers forward	Primers reverse
*β*-Actin	ccaccatgtacccaggcatt	agggtgtaaaacgcagctca
*γ*-Actin	cttacactgcgcttcttgcc	aatgcctgggtacatggtgg
Importin *β*1	gtctctactctgcgcgactc	gctaccactccgtccgtatg
RanBP1	ttaagatgcgtgcaaagctg	gcttcagctccatcattggt
Vimentin	tgaaggaagagatggctcgt	ttgagtgggtgtcaaccaga
CSF1 (ref)	agctggatgatcctgtttgc	tcatggaaagttcggacaca

**Table 2:** RT-PCR validates the presence of importin *β*1, RanBP1, vimentin, *β*-actin, and *γ*-actin in explant lysates and reveals different levels of transcripts in explants and axons. In addition, *γ*-actin is absent from axons (Ct values of >37). Values represent Ct values.

	CSF1	Importin *β*1	RanBP1	Vimentin	*β*-Actin	*γ*-Actin
Axon Ct	30 ± 3	31 ± 3	33 ± 3	27 ± 2	27 ± 3	N/A
Tissue Ct	23 ± 0.2	27 ± 3	31 ± 0.9	25 ± 2	16 ± 0.05	24 ± 0.8

**Table 3:** Western blotting quantification and comparison. Untransformed data is provided for each transcript, as well as reciprocally transformed data for a given transcript, performed to normalize datasets prior to statistical analysis. *∗* indicates significant difference from control.

Transcript	Control	1 hr	3 hrs	6 hrs
Importin *β*1	1.27 ± 0.13	3.45 ± 1.65	1.99 ± 0.42	3.13 ± 0.89
RanBP1	1.08 ± 0.27	1.69 ± 0.73	0.83 ± 0.26	0.94 ± 0.17
Vimentin	0.84 ± 0.12	0.79 ± 0.39	0.83 ± 0.19	0.59 ± 0.20
RT-importin *β*1	0.83 ± 0.09	0.51 ± 0.14^*∗*^	0.62 ± 0.14	0.45 ± 0.12^*∗*^
RT-RanBP1	1.20 ± 0.28	0.89 ± 0.18	1.93 ± 0.68	1.24 ± 0.25
RT-vimentin	1.32 ± 0.25	3.41 ± 1.4	1.48 ± 0.33	2.87 ± 1.03
